# Integrative practices to address stress in nursing professionals during the Covid-19 pandemic: scoping review

**DOI:** 10.1590/1980-220X-REEUSP-2024-0108en

**Published:** 2025-02-03

**Authors:** Girlene Ribeiro da Costa, Márcia Teles de Oliveira Gouveia, Edilaine Cristina da Silva Gherardi-Donato, Priscilla Cavalcante Lima

**Affiliations:** 1 Universidade Federal do Piauí, Teresina, PI, Brazil.; 2 Universidade de São Paulo, Ribeirão Preto, SP, Brazil

**Keywords:** Nursing, Occupational Stress, Complementary Therapies, Coronavirus Infections, Enfermería, Estrés Laboral, Terapias Complementarias, Infecciones por Coronavírus, Enfermagem, Estresse Ocupacional, Terapias Complementares, Infecções por Coronavírus

## Abstract

**Objective:**

To map integrative practices used to address occupational stress in nursing professionals during the Covid-19 pandemic.

**Method:**

This is a scoping review following the recommendations of the JBI Institute and PRISMA Extension for Scoping Reviews (PRISMA-ScR). The search was performed between March and April 2024 in the following databases: MEDLINE, LILACS, BDENF, IBECS via BVS, and CINAHL, SCOPUS and Web of Science via the CAPES Journal Portal. The selection, stratification and organization of the studies were carried out on the Rayyan platform. The results were presented in a synoptic table, with a descriptive and comparative analysis of the findings, highlighting the integrative practices and their evidence of effectiveness.

**Results:**

Eighteen studies were included, most of which were published in the United States in 2021. The most common practices were: mindfulness (16.6%), wobble rooms (11%), psychoeducation (11%), peer support (11%), and nonviolent communication (11%). All were effective in reducing stress, promoting well-being, and improving the quality of life of nursing professionals. Conclusions: The most widely used integrative practices were mindfulness and psychoeducation programs, with encouragement of nonviolent communication, all demonstrating effectiveness as interventions to reduce stress in nursing professionals during the pandemic. These practices emerge as viable and effective strategies to promote the mental health and well-being of these professionals.

## RESUMO

**Objetivo:**

Mapear práticas integrativas utilizadas no enfrentamento do estresse ocupacional em profissionais de enfermagem durante a pandemia da Covid-19.

**Método:**

Trata-se de *scoping review* seguindo as recomendações do Instituto JBI e PRISMA *Extension for Scoping Reviews* (PRISMA-ScR). A busca foi realizada entre março e abril de 2024 nas bases de dados: MEDLINE, LILACS, BDENF, IBECS via BVS, além de CINAHL, SCOPUS e *Web of Science* via Portal de periódicos CAPES. A seleção, estratificação e organização dos estudos foram feitas plataforma *Rayyan*. Os resultados foram apresentados em quadro sinóptico, com uma análise descritiva e comparativa dos achados, destacando as práticas integrativas e suas evidências de eficácia.

**Resultados:**

Foram incluídos 18 estudos, a maioria publicada nos Estados Unidos no ano de 2021. As práticas mais recorrentes foram: *mindfulness* (16,6%), salas de oscilação (11%), psicoeducação (11%), apoio de pares (11%), comunicação não violenta (11%). Todas foram eficazes na redução do estresse, promoção de bem-estar e melhoria da qualidade de vida dos profissionais de enfermagem. Conclusões: As práticas integrativas mais utilizadas foram *mindfulness* e programas de psicoeducação, com estímulo à comunicação não violenta, todas demonstrando efetividade como intervenções para reduzir o estresse em profissionais de enfermagem durante a pandemia. Essas práticas emergem como estratégias viáveis e eficazes para promover a saúde mental e o bem-estar desses profissionais.

## RESUMEN

**Objetivo:**

Mapear las prácticas integradoras utilizadas para afrontar el estrés ocupacional en profesionales de enfermería durante la pandemia de Covid-19.

**Método:**

Esta es una revisión de alcance que sigue las recomendaciones del Instituto JBI y PRISMA Extension for Scoping Reviews (PRISMA-ScR). La búsqueda se realizó entre marzo y abril de 2024 en las bases de datos: MEDLINE, LILACS, BDENF, IBECS vía VHL, además de CINAHL, SCOPUS y Web of Science vía el portal de revistas CAPES. La selección, estratificación y organización de los estudios se realizó en la plataforma Rayyan. Los resultados se presentaron en una tabla sinóptica, con un análisis descriptivo y comparativo de los hallazgos, destacando las prácticas integradoras y su evidencia de efectividad.

**Resultados:**

Se incluyeron 18 estudios, la mayoría publicados en Estados Unidos en 2021. Las prácticas más comunes fueron: mindfulness (16,6%), salas de bienestar (11%), psicoeducación (11%), apoyo entre pares (11%) y comunicación no violenta (11%). Todas resultaron efectivas para reducir el estrés, promover el bienestar y mejorar la calidad de vida de los profesionales de enfermería.

**Conclusiones:**

Las prácticas integradoras más utilizadas fueron los programas de mindfulness y psicoeducación, fomentando la comunicación no violenta, y todas demostraron efectividad como intervenciones para reducir el estrés en los profesionales de enfermería durante la pandemia. Estas prácticas emergen como estrategias viables y efectivas para promover la salud mental y el bienestar de estos profesionales.

## INTRODUCTION

Integrative Health Practices (IHPs) have gained prominence as effective approaches to address occupational stress among nursing professionals, especially during the COVID-19 pandemic^(1,2)^. Unlike traditional approaches, which often focus on drug interventions and reactive treatments, IHPs offer a holistic and preventive perspective, integrating care that considers the physical, emotional, mental, and social aspects of individuals^(3,4)^. This approach is aimed not only at treatment, but also at promoting well-being and quality of life.

Integrative Health Practices have been incorporated into the Unified Health System (SUS) in Brazil in 2021 and encouraged as part of the United Nations (UN) Sustainable Development Goals (SDGs)^(5,6)^ with the aim to improve occupational health. These practices were viable in the context of the pandemic to mitigate the psychological impacts faced by nursing professionals, who became especially vulnerable to insomnia, anxiety, and depression^(2,4)^.

Despite their increasing use, the literature still presents gaps regarding the specific effectiveness of integrative practices in coping with occupational stress in nursing professionals during the COVID-19 pandemic. Although previous reviews have addressed the impact of various interventions on the well-being of these professionals, the application and specific results of IHPs remain insufficiently explored^(2)^.

In view of this scenario, the objective of this study was to map the integrative practices used in nursing professionals for coping with occupational stress during the COVID-19 pandemic, offering a critical analysis of the available evidence and contributing to the development of more effective mental care strategies for these professionals.

## METHODOLOGY

### Type of Study

This is a scoping review, which makes it possible to map the fundamental concepts underpinning a given theme and to assess the expansion, scope and nature of existing research^(7)^.

### Research Question

The research question was developed based on the PCC (Population, Concept, Context) strategy^(7)^, defining P-population: “Nursing professionals”, C-concept: “Integrative practices in stress reduction” and C-context: “COVID/coronavirus”. The strategy resulted in the question: What are the integrative practices used to cope with occupational stress in nursing professionals during the COVID-19 pandemic?

### Methodological Procedure

The recommendations of the JBI manual for scoping reviews were followed in the development of this study, ensuring a transparent and impartial process. The research protocol was registered in the Open Science Framework (OSF) under the DOI identifier: https://doi.org/10.17605/OSF.IO/3GHS7. The strategy is a guide for scoping reviews^(8,9)^ in 9 stages, which were developed between January and April 2024: 1 – development of the research question; 2 – identification of relevant studies; 3 – selection of studies; 4 – mapping of information; 5 –grouping, summary and reporting of results; 6 - consultation; 7 – analysis of evidence; 8 – presentation of results; and 9 – summary of evidence in relation to the objective of the review and conclusions.

The preliminary review was carried out through a refined search in the Cochrane Library, confirming the originality of the topic and the existence of significant literature for the development of the study. Then, a research protocol was developed to define the eligibility criteria. The collection of information with a survey of authors and journals according to theme and year of publication, based on the mnemonic term PCC^(7)^ generated the research question, objective, title and descriptors.

### Selection Criteria

Primary studies, systematic reviews (the inclusion of these was reconsidered to meet the objective of mapping evidence, as suggested by the JBI manual), reflective reviews, guidelines, directives, websites, letters, descriptive reports and official communications from government institutions, without time limits, in Portuguese, English and Spanish were included. Studies that addressed pharmacological strategies for managing occupational stress, contemplated only other professional categories and were unrelated to the theme were excluded.

### Search Strategies

Once the research question was defined, the literary corpus to be analyzed was surveyed. The Boolean operators AND and OR were used to cross-reference the controlled and uncontrolled descriptors indexed in the Health Sciences Descriptors (DECs) and Medical Subject Headings (MeSH).

Data collection was carried out between March and April 2024 through the insertion of distinct search strategies appropriate to each database, including: National Library of Medicine (MEDLINE), Latin American and Caribbean Literature in Health Sciences (LILACS), Nursing Database (BDENF), Spanish Bibliographic Index of the Health Sciences (IBECS), accessed via BVS, and Web of Science, Cumulative Index to Nursing and Allied Health Literature (CINAHL), in addition to Scopus, accessed via the CAPES Portal (Fundação Coordenação de Aperfeiçoamento de Pessoal de Nível Superior), through identification in the Federated Academic Community (CAFe). The descriptors and strategies are described in Chart 1.

**Chart 1:** Search strategies in Portuguese, Spanish and English used in the databases searched – Teresina, PI, Brazil, 2024.

Database	Search strategies
**BVS – LILACS, BDENF e IBECS (Decs)**	((mh:(“Enfermeiras e Enfermeiros”)) *OR* (“Enfermeiras e Enfermeiros”) *OR* (Enfermeira) *OR* (Enfermeiro) *OR* (*Nurses*) *OR* (“*Registered Nurses*”) *OR* (“*Enfermeras y Enfermeros*”) *OR* (“*Enfermera*”) *OR* (“*Enfermero*”)) *AND* ((mh:(“Estresse Ocupacional”)) *OR* (“estresse ocupacional”) *OR* (“Estresse Profissional”) *OR* (“Estresse Relacionado ao Ambiente de Trabalho”) *OR* (“Estresse Relacionado ao Trabalho”) *OR* (“Estresse Relacionado à Profissão”) *OR* (“Estresse do Ambiente de Trabalho”) *OR* (“Estresses Relacionados ao Ambiente de Trabalho”) *OR* (“Estresses Relacionados à Profissão”) *OR* (“Estresses do Ambiente de Trabalho”) *OR* (“*Occupational Stress*”) *OR* (“*Estrés Laboral*”) *OR* (“Terapia de redução de Estresse”) *OR* (“*Job Stress*”) *OR* (“*Job Stresses*”) *OR* (“*Job related Stress*”) *OR* (“*Occupational Stresses*”) *OR* (“*Work Place Stress*”) *OR* (“*Estrés Profesional*”) *OR* (“*Estrés Relacionado a la Profesión*”) *OR* (“*Estrés Relacionado con el Trabajo*”)) *AND* ((mh:(“Infecções por Coronavírus”)) *OR* (“Infecções por Coronavírus”) *OR* (Covid-19) *OR* (“Covid 19”) *OR* (“Doença pelo Novo Coronavírus (2019-nCoV)”) *OR* (“Infecção pelo Coronavírus 2019-nCoV”) *OR* (“Infecções por Coronavírus”) *OR* (Coronavírus) *OR* (“*Coronavirus Infections*”) *OR* (“2019 *novel coronavirus Epidemic*”) *OR* (“2019 *novel coronavirus Pandemic*”) *OR* (“2019-nCoV *Pandemic*”) *OR* (“*Coronavirus Infection*”) *OR* (“Infecções por Coronavírus”) *OR* (“*Enfermedad por Coronavirus* 2019-nCoV”) *OR* (“*Infección por Coronavirus* 2019-nCoV”) *OR* (“*Infección por el Coronavirus* 2019-nCoV”) *OR* (“*Infección por el Nuevo Coronavirus* (2019-nCoV)”))
**MEDLINE via PubMed (MeSH)**	(“*Nurses*”[MeSH *Terms*] *OR* “*Nurses*”[*All Fields*] *OR* “*nurse*”[*All Fields*] *OR* “*nursing personnel*”[*All Fields*] *OR* “*registered nurses*”[*All Fields*] *OR* “*registered nurse*”[*All Fields*]) *AND* (“*Occupational Stress*”[MeSH *Terms*] *OR* “*Occupational Stress*”[*All Fields*] *OR* “*occupational stresses*”[*All Fields*] *OR* “*job stress*”[*All Fields*] *OR* “*work related stress*”[*All Fields*] *OR* “*workplace stress*”[*All Fields*] *OR* “*work place stress*”[*All Fields*] *OR* “*work place stresses*”[*All Fields*] *OR* “*professional stress*”[*All Fields*] *OR* “*job related stress*”[*All Fields*]) *AND* (“Covid-19”[*MeSH Terms*] *OR* “Covid-19”[*All Fields*] *OR* “2019 *ncov infection*”[*All Fields*] *OR* “*coronavirus disease* 19”[*All Fields*] *OR* “2019 *novel coronavirus disease*”[*All Fields*] *OR* “2019 *novel coronavirus infection*”[*All Fields*] *OR* “2019 *ncov disease*”[*All Fields*] *OR* “Covid-19”[*All Fields*] *OR* “*coronavirus disease* 2019”[*All Fields*] *OR* “*sars coronavirus 2 infection*”[*All Fields*] *OR* “*sars cov 2 infection*”[*All Fields*])
**CINAHL (List CINAHL)**	((MH “*Nurses*”) *OR* “*Nurses*” *OR* “*Nurse*” *OR* “*Nursing Personnel*” *OR* (MH “*Registered Nurses*”) *OR* “*Registered Nurses*” *OR* “*Registered Nurse*”) *AND* ((MH “*Stress*, *Occupational*”) *OR* “*Occupational Stress*” *OR* “*Occupational Stresses*” *OR* “*Job Stress*” *OR* “*Work related Stress*” *OR* “*Workplace Stress*” *OR* “*Work Place Stress*” *OR* “*Work Place Stresses*” *OR* “*Professional Stress*” *OR* “*Job related Stress*”) *AND* ((MH “Covid-19”) *OR* “Covid-19” *OR* “Covid-19” *OR* “2019 *nCoV Infection*” *OR* “*Coronavirus Disease* 19” *OR* “2019 *Novel Coronavirus Disease*” *OR* “2019 *Novel Coronavirus Infection*” *OR* “2019 *nCoV Disease*” *OR* “Covid-19” *OR* “SARS *Coronavirus 2 Infection*” *OR* “SARS CoV 2 *Infection*”)
**Scopus (MeSH)**	((*TITLE-ABS-KEY*(“*Nurses*”) *OR TITLE-ABS-KEY*(“*Nurse*”) *OR TITLE-ABS-KEY*(“Nursing *Personnel*”) *OR TITLE-ABS-KEY*(“*Registered Nurses*”) *OR TITLE-ABS-KEY*(“*Registered Nurse*”))) *AND*((*TITLE-ABS-KEY*(“*Occupational Stress*”) *OR TITLE-ABS-KEY*(“*Occupational Stresses*”) *OR TITLE-ABS-KEY*(“*Job Stress*”) OR TITLE-ABS-*KEY*(“*Work related Stress*”) *OR TITLE-ABS-KEY*(“*Workplace Stress*”) *OR TITLE-ABS-KEY*(“*Work Place Stress*”) *OR TITLE-ABS-KEY*(“*Work Place Stresses*”) *OR TITLE-ABS-KEY*(“*Professional Stress*”) *OR TITLE-ABS-KEY*(“*Job related Stress*”))) *AND*((*TITLE-ABS-KEY*(“Covid-19”) *OR TITLE-ABS-KEY*(“Covid 19”) *OR TITLE-ABS-KEY*(“2019 nCoV *Infection*”) *OR TITLE-ABS-KEY*(“*Coronavirus Disease* 19”) *OR TITLE-ABS-KEY*(“2019 *Novel Coronavirus Disease*”) *OR TITLE-ABS-KEY*(“2019 *Novel Coronavirus Infection*”) *OR TITLE-ABS-KEY*(“2019 *nCoV Disease*”) *OR TITLE-ABS-KEY*(Covid-19) *OR TITLE-ABS-KEY*(“*Coronavirus Disease* 2019”) *OR TITLE-ABS-KEY*(“*SARS Coronavirus 2 Infection*”) *OR TITLE-ABS-KEY*(“SARS CoV 2 *Infection*”)))
Web of Science **(MeSH)**	(TS = (“*Nurses*”) *OR* TS = (“*Nurse*”) *OR* TS = (“*Nursing Personnel*”) *OR* TS = (“*Registered Nurses*”) *OR* TS = (“*Registered Nurse*”)) *AND* (TS = (“*Occupational Stress*”) *OR* TS = (“*Occupational Stresses*”) *OR* TS = (“*Job* Stress”) *OR* TS = (“*Work related Stress*”) *OR* TS = (“*Workplace Stress*”) *OR* TS = (“*Work Place Stress*”) *OR* TS = (“*Work Place Stresses*”) *OR* TS = (“*Professional Stress*”) *OR* TS = (“*Job related Stress*”)) *AND* (TS = (“Covid-19”) *OR* TS = (“Covid-19”) *OR* TS = (“2019 *nCoV Infection*”) *OR* TS = (“*Coronavirus Disease* 19”) *OR* TS = (“2019 *Novel Coronavirus Disease*”) *OR* TS = (“2019 *Novel Coronavirus Infection*”) *OR* TS = (“2019 *nCoV Disease*”) *OR* TS = (“Covid-19”) *OR* TS = (“*Coronavirus Disease* 2019”) *OR* TS = (“SARS *Coronavirus 2 Infection*”) *OR* TS = (“SARS CoV 2 *Infection*”))

Legend: National Library of Medicine (MEDLINE), Latin American and Caribbean Health Sciences Literature (LILACS), Nursing Database (BDENF), Spanish Bibliographic Index on Health Sciences (IBECS), BVS-Virtual Library and Web of Science, Cumulative Index to Nursing and Allied Health Literature (CINAHL). **Source:** Prepared by the authors.

### Selection of Studies

After the search, the files with the studies were exported to the Rayyan QCRI^®^ centralization tool, where duplicates were removed. Then, peer review was carried out with two independent researchers, each one making an individual selection of the eligible studies. At the end, the researchers met to resolve disagreements and review the eligibility criteria.

The PRISMA-ScR extension standard for conducting Scoping Reviews (PRISMA-ScRO) was followed in the process of systematic organization of the sample for development of the study, analysis of studies, and construction of the results^(10)^.

### Data Extraction and Analysis

The literature on the topic, including the legal framework on IHPs mentioned above, was used in the theoretical discussion. An instrument developed by the reviewers, based on the model available in the JBI manual and containing objective, year, country and method, was considered in data extraction^(8,9)^. The results were presented in a synoptic table with a descriptive and comparative analysis of the findings with the global scientific literature.

## RESULTS

**Figure 1:**
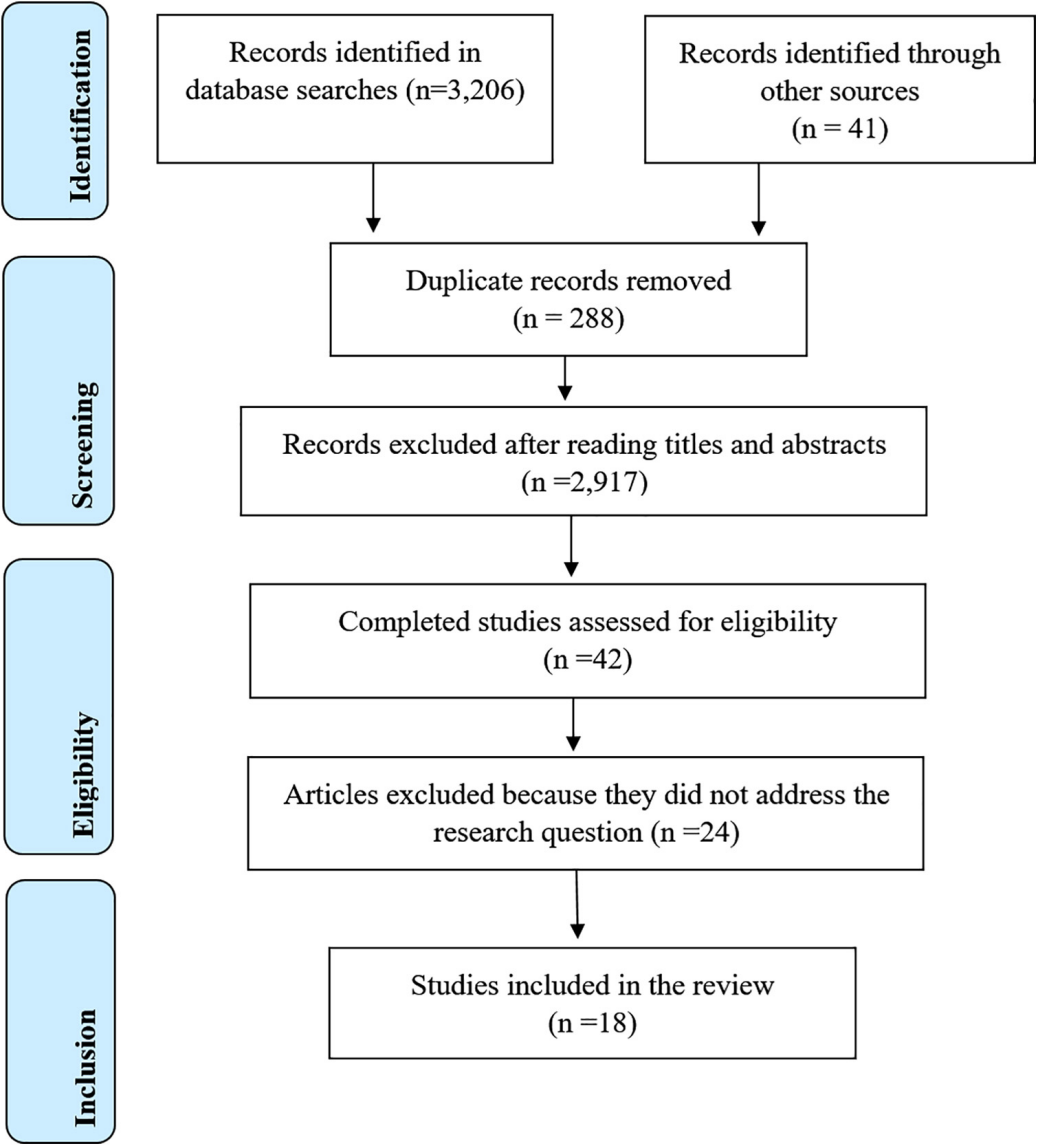
PRISMA-ScR flowchart of selection of studies and process of inclusion in the scoping review. Teresina, PI, Brazil, 2024.

Source: Prepared by the authors.

The search strategies retrieved 3,206 records, and 41 additional records were identified by manual searching of other sources. After excluding 288 duplicate references, 2,959 articles were analyzed for title and abstract, applying the previously established eligibility criteria. From this analysis, 42 studies were selected for full reading, and 24 of these were excluded because they did not directly answer the research question, resulting in a final sample of 18 studies eligible for inclusion in the review (Figure 1).

Chart 2 provides a summary and characterization of the findings. Most of the selected studies were conducted in the United States of America (USA) (22%, n = 24), with a predominance of publications in 2021 (61%, n = 11). Regarding the design, most were intervention studies (89%, n = 16).

**Chart 2:** Characterization of the studies selected in the scoping review regarding year, country, objective, method, measurement instrument, integrative practice and outcome – Teresina, PI, Brazil, 2024.

ID*/Year/ Country	Objective	Method/Measurement instrument	Integrative practice and outcome
A1^(11)^ 2021 United Kingdom	To support staff who perceive themselves as struggling with pandemic-related pressures in promoting and maintaining well-being.	Letter to the Editor Descriptive, exploratory	“Staff Wobble and Relaxation Rooms” ✓ Participating and listening to colleagues are experienced as validation. Significant levels of anxious anticipation about possible scenarios of patient management difficulties and loss experiences were observed.
A2^(12)^ 2021 USA	To evaluate a stress reduction strategy, a web-based Mantra Repetition Program, for nurses caring for hospitalized veterans.	Mixed method Schmidt Perception of Nursing Care Survey and Presence of Nursing Scale	Mantra Repetition Program (MRP) ✓ Nurses perceived greater mindfulness, compassion satisfaction, spiritual well-being and less burnout.
A3^(13)^ 2021 United Kingdom	To highlight the importance of a well-being champion in the intensive care unit who promotes self-reflective practice and self-care to protect staff well-being.	Commentary	Self-reflective practice “Look, Listen and Connect”: Psychological First Aid. ✓ The practice promoted self-care and compassion, cultivating a positive mindset, resilience, compassion for oneself and others, and well-being.
A4^(14)^ 2020 Canada	To proactively support and protect direct care nurses from the adverse mental health effects of COVID-19.	Descriptive	The “wobble room” with well-being intervention. ✓ Staff were able to understand how the pandemic was affecting them and created a “new normal” in relation to personal safety and team cohesion.
A5^(15)^ 2021 USA	To assess the effectiveness of intentional self-care practices on nurse burnout and the workplace by measuring job satisfaction and teamwork among nurses	Retrospective study Nurse Survey with Practice Environment Scale	Mindfulness-based self-care practices (Project7 Mindfulness Pledge) ✓ Nurses on an inpatient unit who implemented Project 7 had higher job satisfaction compared nurses in units where the project was not implemented
A6^(16)^ 2021 Japan	To identify individuals at high risk for mental health issues and provide brief psychotherapy to them	Letter to the Editor Preliminary non-randomized study Kessler Psychological Distress Scale	Individual psychotherapy for nurses at high risk of stress using psychological first aid. ✓ Improvement in psychological distress in nurses identified as high risk who received psychotherapy, compared to those who did not.
A7^(17)^ 2021 France	To assess the effectiveness of remote EMDR treatment for healthcare workers who were distressed by their work with individuals with COVID-19	Pilot, quasi-experimental study Hospital Depression Anxiety Scale and level of perceived distress	Eye movement desensitization and reprocessing therapy, remote psychotherapy. ✓ Improvement in emotional state and a decrease in perceived distress.
A8^(18)^ 2020 England	To assess facility utilization and gather information on staff well-being and employee feedback regarding this provision	Service use monitoring study Warwick Edinburgh Mental Wellbeing measure; Utrecht Work dedication subscale. Engagement scale	Wellness Centers for 17 weeks ✓ Well-being was higher in those who accessed a wellness center. Work engagement and job satisfaction were high.
A9^(19)^ 2021 France	To promote calming, soothing, and support to staff members who have been overwhelmed for several weeks to help them with coping	Brief descriptive, qualitative report.	Program and space of relaxation and support for hospital caregivers by hospital caregivers, the Port Royal Bulle (the Bubble)
A10^(20)^ 2021 Brazil	To reflect on the use of empathic listening as an embracement strategy for nursing professionals in coping with challenges during the novel coronavirus pandemic	Reflection study	Non-violent communication and empathic listening. ✓ Professional satisfaction and well-being, self-care, quality of life
A11^(21)^ 2020 Spain	To describe a rapid, mindfulness-based, on-site intervention and explore its feasibility, utility, and safety for frontline healthcare workers in the midst of the COVID-19 outbreak	Exploratory study with post-intervention evaluation Questionnaire developed: session attendance and perceived usefulness in reducing current stress	Mindfulness practices ✓ The sessions were perceived as being useful, with an average rating of 8.4 on a 0-10 scale. Only three people (2%) reported a minor adverse effect (increased anxiety or dizziness)
A12^(22)^ 2020 China	To investigate the difference in work stress between nurses in emergency services and non-emergency services; to propose a bidirectional narrative nursing intervention	Bidirectional narrative method Basic situation questionnaire form; work pressure scale	Two-way problem-solving narrative ✓ Reduced nurses’ work pressure, providing a basis for work coordination
A13^(23)^ 2021 USA	To support caregivers during COVID-19 through the peer support model	Letter to the editor Report Descriptive study	Peer supporters are trained to support their colleagues in the face of a critical event ✓ Reduction of the three components of burnout: emotional exhaustion, depersonalization and reduced effectiveness
A14^(24)^ 2021 Turkey	To investigate the effectiveness of a brief online form of Emotional Freedom Techniques in preventing stress, anxiety and burnout in nurses involved in the treatment of patients with COVID-19	Randomized clinical trial Subjective units of distress scale, State-Trait Anxiety Inventory and Burnout scale	The Emotional Freedom Techniques online (EFT) ✓ A single online group session reduced stress, anxiety and burnout levels in nurses treating COVID-19
A15^(25)^ 2021 USA	To improve the health and well-being of frontline nurses during the COVID-19 pandemic	Descriptive, randomized study	Wellbeing Partners Program: peer support, physical activity, healthy eating, sleep and stress management, sustainable solutions, well-being and healthy lifestyle coaching sessions ✓ 98% of nurses shared that the program helped them engage in self-care and well-being, and 94.7% agreed that the program contributed to improved mental and physical health
A16^(26)^ 2022 Turkey	To investigate the effect of mindfulness-based breathing and music therapy on stress, work-related strain and levels of psychological well-being of nurses who provided care to COVID-19 patients	Randomized Case-Control Trial The State Anxiety Inventory, Psychological Well-Being Scale, Work-Related Strain Scale.	Mindfulness-based breathing techniques and music therapy ✓ decreased work-related stress and tension and increased psychological well-being in nurses of COVID-19
A17^(27)^ 2023 Ghana	To compare the perceived differences in Quality of Work-Life between nurses and coping strategies used in COVID-19	Descriptive, cross-sectional, multistage sampling technique Work-Related Quality of Life Scale (WRQoL)	Work-family segmentation, search for care, open communication and recreational activities ✓ Most nurses who used the techniques reported greater ability to cope with challenges
A18^(28)^ 2023 Jordan	To evaluate the effectiveness of a psychoeducational program for COVID-19 nurses	Randomized clinical trial Nursing Stress Scale [NSS] and Brief Coping Scale	Psychoeducational program: six sessions of stress management methods, relaxation and breathing exercises; positive self-talk; therapy of revealing happy thoughts, and assertiveness training. There was a reduction in stress in the intervention group

**Source:** Prepared by the authors, 2024.

The most frequently used interventions included mindfulness (16.6%, n = 3), wobble rooms (11%, n = 2), communication (11%, n = 2), relaxation space (11%, n = 2), pseudo-education (11%, n = 2), peer support (11%, n = 2), and eye movement desensitization and reprocessing therapy (5.5%, n = 1), and mantra repetition program (5.5%, n = 1). The effectiveness of these interventions was assessed based on before-and-after studies using validated scales for stress (27.7%, n = 5), quality of life and well-being (22.2%, n = 4), anxiety (11%, n = 2), depression (5.5%, n = 1), and distress (5.5%, n = 1).

In some studies, the scales used to measure the highlighted variables could be identified, such as: Professional Quality of Life Scale, Nursing Presence Scale^(12)^, Mindfulness Attention and Awareness Scale, Functional Assessment of Spiritual Well-Being of Chronic Illness Therapy Scale, Nurse Survey with Practice Environment Scale^(15)^, Kessler Psychological Distress Scale reduced scale (K6)^(16)^, Hospital Anxiety Depression Scale and the level of perceived disturbance^(17)^, Warwick Edinburgh Mental Well Being Scale (WEMWBS), Utrecht Work Dedication subscale, Engagement Scale^(18)^, Basic Situation Questionnaire Form, Chinese Nurses Work Pressure Scale^(22)^, Subjective Units of Distress Scale, State-Trait Anxiety Inventory and Burnout Scale^(24)^, The State Anxiety Inventory, Psychological Well-Being Scale, Work-Related Strain Scale^(26)^, Work-Related Quality of Life Scale (WRQoL)^(27)^, Nursing Stress Scale [NSS] and Brief COPE Scale^(28)^.

## DISCUSSION

This scoping review allowed mapping the main integrative practices used to cope with occupational stress among nursing professionals during the COVID-19 pandemic, as well as the main suggestions and recommendations to reduce the effects of stress on the mental health of health professionals.

Most of the published studies were from the USA^(12,15,25,27)^ and randomized^(24,26,28)^. This predominance may be attributed to the large volume of scientific publications in the country and the high number of COVID-19 cases, compared to other locations. Randomized studies were more common and are crucial to assess the effectiveness of health interventions. The quality of these studies is essential^(9)^, and the articles reviewed presented significant levels of scientific evidence.

Some institutions mentioned in the studies hired teams to create or develop rest spaces for employees. Scholars^(11,14,19)^ have highlighted the creation of wobble rooms, referred to as “swing rooms,” “break rooms,” “staff relaxation rooms,” and “wellness centers.” These facilities have gained greater importance during the COVID-19 pandemic due to increased stress levels and work overload^(12,16,21,23)^. Their goal was to provide support for the health of professionals, and they have resulted in reduced stress and improved well-being for frontline workers. The rooms have been described as safe places off the base/ward, readily available 24 hours a day, offering comfort, distraction (music/reading materials), food, relaxation, facilitated reflection sessions, exercise, and peer support^(11)^.

One of the studies combined strategies for self-management of stress among nurses during COVID-19, including the six-week Psychoeducational Program implemented in Jordan. The practice included training in progressive relaxation techniques, time management, promotion of laughter, deep breathing exercises, among others^(28)^. At the same time, music therapy also stood out in Turkey as an effective method for reducing stress and tension among nurses caring for patients infected with COVID-19, promoting a significant increase in psychological well-being. This strategy combined music with mindfulness and breathing techniques^(26)^.

Despite the initiatives, not all employees participate in the relaxation moments offered by the company. A study^(18)^ detailed some justifications reported by professionals who did not participate in the wellness centers, namely: the breaks were not long enough (27.8%); the center was too far from the workplace (26.4%); they could not take a break to participate (21%); preferred a private space than a public one (18.8%); did not feel the need (16.3%); worked from home office (12.3%); were not aware of the centers or did not know whether non-clinical staff could attend (10.4%); did not have enough space or seating in the center (4.1%); did not have a friend available (1.9%). Thus, it is important to engage these professionals with alternative and attractive approaches to facilitate the relaxation process and improve their quality of life.

Some possible strategies were described by the authors of a study carried out in Wuhan^(22)^. Another nursing staff considered psychotherapy, assertive communication, listening to music, reading a book, video calling friends or family, accessing appropriate personal protection, resting appropriately and talking about feelings and emotions as significant. These strategies helped to cope with psychological stress^(27)^. Studies have shown that social support, in particular peer support, limits the effect of trauma exposure on stress symptoms, protecting against depression, anxiety, psychological distress and burnout^(18)^.

In a study^(24)^ conducted with nurses working in the COVID-19 sector in Turkey, professionals were described as vulnerable to emotions such as fear and anxiety due to fatigue, discomfort and helplessness related to high-intensity work. In this group, Emotional Freedom Techniques (EFT) were effective in preventing stress, anxiety and burnout. A single online group session reduced levels of stress, anxiety and burnout in nurses treating COVID-19. The reductions in stress, anxiety and burnout reached levels of statistical significance for the intervention group. In addition, more than half of nurses (68%) reported being motivated and recognized the need to focus on self-care and well-being.

Nursing professionals should seek self-management of their own mental health, as exemplified by African nurses in relation to individual resilience strategies during the pandemic, such as family support, open communication and recreational activities, which positively reflected on the quality of life of these professionals^(27)^.

The importance of taking care of basic needs, exercising and maintaining a healthy and balanced diet was also mentioned. Diaphragmatic breathing exercises and stretching activities performed every two hours contribute to maintaining good brain health, stimulating the vagal nerve and activating the parasympathetic system. Even performed briefly, for periods of four or five minutes, these exercises have shown effectiveness in controlling symptoms of anxiety and stress^(22)^.

In addition to supporting nurses during the COVID-19 pandemic, the peer support model has been recognized as a crucial component for institutional well-being. These interventions helped to minimize the three components of exhaustion – emotional exhaustion, depersonalization and reduced effectiveness – that make up the burnout syndrome. Simple active listening has been shown to be positively beneficial, establishing relationships of trust through encouraging phrases and invitations to reflect on emotions, normalizing and summarizing concerns^(23)^.

Sometimes, psychological distress and stress may be nonexistent when professionals have high resilience in coping, or even in cases of underreporting. In a study conducted in China in 2020, even though no statistically significant difference between emergency room and non-emergency room nurses (p > 0.05) has been found, a significant difference in the source of pressure at work (p < 0.05) was identified. After a month of narrative intervention in Nursing, no significant changes were observed in time distribution, workload, work environment, patient care, management, and interpersonal relationships (p > 0.05)^(22)^. Practical methods to reduce stressors among professionals should be developed, highlighting the willingness to invest in self-direction practices in care, strengthening autonomy and creativity.

In Spain, mindfulness practices lasting an average of 5–10 minutes, twice a day, delivered by psychiatrists, psychologists, and mental health nurses were used in a post-intervention approach. Each session focused on three elements: (1) breathing, body parts such as hands or feet, or surrounding sounds; (2) conscious movement through gentle yoga-like stretching exercises performed standing or sitting, adapted to any physical condition; and (3) compassion through kind and embracing language and attitudes. Nurses rated the intervention as helpful for stress, with an average of 8.4 on a 0–10 scale^(21)^.

French researchers developed a relaxation space in the hospital environment to support professionals, offering activities that facilitated decompression and relaxation. The environment included a warm welcome, attention, listening, empathetic support, and the opportunity to participate in relaxing or low-impact physical activities. In the Bubble Program, structured in four areas, the following were created: (1) embracement and space for conversation and reminders about hygiene; (2) changing room for storing belongings and washing hands; (3) wellness room for relaxation and physical activities; and (4) living room for conversation and listening. Most participants were nurses, making up 57% of the total^(19)^.

In Japan, individual psychotherapy was promoted for nurses at high risk of mental illness. Psychological first aid sessions lasting 30 to 60 minutes were provided, focusing on general conditions of daily life and interpersonal relationships. There was a significant improvement in psychological distress and sleep and appetite disorders, although there were no significant effects on alcohol misuse^(16)^.

Burnout is especially relevant in the current health climate, with frontline nurses facing an increased workload and multiple psychosocial stressors during the COVID-19 pandemic. The Project7 Mindfulness Pledge^©^ is an accessible, voluntary tool that has helped nurses reduce burnout without requiring significant time commitments^(15)^.

Peer support has proven effective in supporting workplace well-being. Staff well-being requires an organizational, unit, and individual approach to encourage self-care and compassion while cultivating a positive mindset^(13)^.

The Mantra Repetition Program (MRP) delivered by nursing practitioners increased mindfulness, compassion, satisfaction, spiritual well-being, and nursing presence, while reducing burnout. The I-MRP was delivered in six one-hour sessions over three months, suggesting that practitioners are willing to learn portable stress reduction techniques^(12)^.

Empathic listening, as an extension of nonviolent communication, is a relevant strategy for minimizing stressors at work and their repercussions, such as violence, bullying, turnover, and absenteeism. In addition, it promotes a culture of peace and strengthens nursing professionals during the pandemic^(20)^.

The sample, consisting of nursing professionals, was sensitized to promote and preserve the health of professionals working during the COVID-19 pandemic. This included changes in the structural environment, psychoeducation programs for self-care, and meditation, self-reflection, and self-care strategies. Specific programs for stress reduction, such as the wobble room^(11,14)^ and the wellness center^(18)^, were implemented, with emphasis on mindfulness, the most adopted technique^(15,21,26)^.

In addition, the adoption of proven effective non-pharmacological practices such as the practice of repeating mantras^(12)^, self-reflective practice^(13)^, and Eye Movement Desensitization and Reprocessing Therapy (EMDR)^(17)^ was discussed. Instruments to measure stress, well-being, quality of life, anxiety, depression, distress, pressure at work, among others, were applied to measure the effectiveness of the interventions.

The practice of mindfulness in sessions^(15,21)^ and artistic-cultural approaches, such as music therapy^(26)^, led the interventions. Communication^(20,27)^ and active listening among peers^(23,25)^ were also highlighted in coping with mental illness among nursing professionals in the face of adversities brought about by the COVID-19 pandemic.

In summary, the use of integrative practices has proven to be valuable as a coping strategy, especially psychological, and contributes to the future management of adversities experienced in the daily lives of nursing professionals. Mindfulness stands out as an accessible and viable practice. The use of instruments to measure stress, depression, anxiety, quality of life, and other variables is also important to validate such practices, demonstrating the scientific efficacy of sensitive strategies. This study reinforces the need for new randomized studies to evaluate the efficacy of therapeutic interventions in reducing stress in health professionals, especially nurses.

A limitation of this study is the fact that most of the articles analyzed evaluated the impact of integrative practices using randomized quantitative methods. Despite their high level of evidence, such methods demonstrate gaps in the qualitative analysis of subjective and abstract variables, such as stress and mental illness. The theoretical awareness of the researchers was an alternative to overcome this gap in order to present the synthesis of the findings with greater reach for the readers.

## CONCLUSION

The integrative practices used to cope with occupational stress in nursing professionals during the COVID-19 pandemic included mindfulness, psychoeducation programs for professionals and their peers, encouragement of nonviolent communication and active listening, changes in the structural environment with the creation of wobble rooms and wellness centers, strategies for self-care and self-reflection, meditation with mantras, among others. For the most part, the interventions had their effectiveness attested by scales for measuring stress and symptoms of depression, anxiety, and distress.

The results point to the need for a multidimensional approach to stress, especially considering strategies conducted by the individual. The recovery of mental health converges with the prevention of future illnesses, in a low-cost scenario and primary care. As described in the literature, it is important to provide immediate post-trauma support, which should be concrete, non-intrusive and adapted to the needs and expectations of the people to whom it is offered. This review also suggests that evidence-based conscious self-care practices need to be reinforced to impact occupational illness.

Conducting systematic reviews and studies based on primary data with a qualitative design and group sampling is another suggestion to validate the effectiveness of integrative practices in coping with stress, depression and anxiety.

## References

[1] Pereira EC, Souza GCD, Schveitzer MC (2022). Práticas Integrativas e Complementares ofertadas pela enfermagem na Atenção Primária à Saúde. Saúde Debate.

[2] Costa NNG, Servo MLS, Figueredo WN (2022). COVID-19 and the occupational stress experienced by health professionals in the hospital context: integrative review. Rev Bras Enferm.

[3] National Academies of Sciences, Engineering, and Medicine (2021). The Future of Nursing 2020-2030: charting a path to achieve health equity – 10, Supporting the Health and Professional Well-Being of Nurses.

[4] Sriharan A, Ratnapalan S, Tricco AC, Lupea D (2021). Women in health care experiencing occupational stress and burnout during Covid-19: a review. BMJ Open.

[5] (2020). Informe de evidência clínica em práticas integrativas e complementares em saúde n° 01/2021 Saúde do Trabalhador. Brasília.

[6] (2017). Relatório nacional voluntário sobre os Objetivos de Desenvolvimento Sustentável [Internet].

[7] Peters MDJ, Godfrey CM, Khalil H, McInerney P, Parker D, Soares CB (2015). Guidance for conducting systematic scoping reviews. Int J Evid-Based Healthc.

[8] Peters MD, Godfrey C, McInerney P, Baldini Soares C, Khalil H, Parker D, Aromataris E, Munn Z (2017). Joanna Briggs Institute Reviewer’s Manual.

[9] Peters MD, Marnie C, Tricco A, Pollock D, Munn Z, Alexander L (2020). Updated methodological guidance for the conduct of scoping reviews. JBI Evidence Synthesis.

[10] Tricco AC, Lillie E, Zarin W, O’Brien KK, Colquhoun H, Levac D (2018). PRISMA Extension for Scoping Reviews (PRISMA-ScR): checklist and explanation. Ann Intern Med.

[11] Veitch P, Richardson K (2021). Nurses need support during Covid-19 pandemic. J Psychiatr Ment Health Nurs.

[12] Kostovich CT, Bormann JE, Gonzalez B, Hansbrough W, Kelly B, Collins EG (2021). Being present: examining the efficacy of an Internet Mantram Program on RN-delivered patient-centered care. Nurs Outlook.

[13] Wharton C, Kotera Y, Brennan S (2021). A well-being champion and the role of self-reflective practice for ICU nurses during Covid-19 and beyond. Nurs Crit Care.

[14] Gurney L, Lockington J, Quinn L, MacPhee M (2020). Why do we need wobble rooms during Covid-19?. Nurs Leadersh (Tor Ont).

[15] Monroe C, Loresto F, Horton-Deutsch S, Kleiner C, Eron K, Varney R (2021). The value of intentional self-care practices: the effects of mindfulness on improving job satisfaction, teamwork, and workplace environments. Arch Psychiatr Nurs.

[16] Kameno Y, Hanada A, Asai D, Naito Y, Kuwabara H, Enomoto N (2021). Individual psychotherapy using psychological first aid for frontline nurses at high risk of psychological distress during the Covid-19 pandemic. Psychiatry Clin Neurosci.

[17] Tarquinio C, Brennstuhl MJ, Rydberg JA, Bassan F, Peter L, Tarquinio CL (2021). EMDR in telemental health counseling for healthcare workers caring for Covid-19 patients: a pilot study. Issues Ment Health Nurs.

[18] Blake H, Yildirim M, Wood B, Knowles S, Mancini H, Coyne E (2020). Covid-Well: evaluation of the implementation of supported wellbeing centres for hospital employees during the Covid-19 pandemic. Int J Environ Res Public Health.

[19] Lefèvre H, Stheneur C, Cardin C, Fourcade L, Fourmaux C, Tordjman E (2021). The bulle: support and prevention of psychological decompensation of health care workers during the trauma of the covid-19 epidemic. J Pain Symptom Manage.

[20] Tobase L, Cardoso SH, Rodrigues RTF, Peres HHC (2021). Empathic listening: welcoming strategy for nursing Professional in coping with with the coronavirus pandemic. Rev Bras Enferm.

[21] Rodriguez-Vega B, Palao Á, Muñoz-Sanjose A, Torrijos M, Aguirre P, Fernández A (2020). Implementation of a mindfulness-based crisis intervention for frontline healthcare workers during the Covid-19 outbreak in a Public General Hospital in Madrid, Spain. Front Psychiatry.

[22] Yanjun Z, Xueqing X (2020). Application of two-way narrative intervention in emergency department nurses under the novel coronavirus pneumonia epidemic. Acta Med Mediter.

[23] Pelt MV, Morris T, Lilly AC, Pian-Smith MCM, Karasik L, Pelt FV (2021). Supporting caregivers during Covid-19: transforming compassionate care from a way of doing to being. AANA J.

[24] Dincer B, Inangil D (2021). The effect of Emotional Freedom Techniques on nurses’ stress, anxiety, and burnout levels during the Covid-19 pandemic: a randomized controlled trial. Explore (NY).

[25] Teall AM, Mazurek B (2021). An innovative wellness partner program to support the health and well-being of nurses during the Covid-19 pandemic: implementation and outcomes. Nurs Adm.

[26] Yıldırım D, Çirisş Yıldız C (2022). The effect of mindfulness-based breathing and music therapy practice on nurses’ stress, work-related strain, and psychological well-being during the covid-19 pandemic: a randomized controlled trial. Holist Nurs Pract.

[27] Poku CA, Bayuo J, Mensah E, Bam V (2023). Quality of work-life and coping strategies of nurse educators and clinicians in COVID: A cross-sectional study. Nurs Open.

[28] Alkhawaldeh JM (2023). Psychoeducational interventional programme during the Covid-19 pandemic for nurses with severe occupational stress: a randomized controlled trial. Int J Nurs Pract.

